# Aortoesophageal fistula following emergency thoracic endovascular aortic repair: 2 case reports and brief review

**DOI:** 10.1097/MD.0000000000045547

**Published:** 2025-11-21

**Authors:** Zhi Wen, Zheng Wang, Jianqiang Wu, Mingwu Tian, Changxue Wu

**Affiliations:** a Cardiothoracic and Vascular Surgery, Deyang People’s Hospital, Deyang, Sichuan Province, China.

**Keywords:** aortic pseudoaneurysm, aortoesophageal fistula, etiopathogenic mechanisms, thoracic endovascular aortic repair, true thoracic aortic aneurysm

## Abstract

**Rationale:**

Aortoesophageal fistula (AEF) following thoracic endovascular aortic repair (TEVAR) is a rare but highly fatal complication. Despite successful initial repair, factors such as large aneurysms and mediastinal hematoma may lead to persistent esophageal compression and ischemia, culminating in delayed AEF. This report presents 2 fatal cases to highlight the diagnostic challenges and management pitfalls of this condition, underscoring the need for increased vigilance in high-risk patients even after successful procedures.

**Patient concerns:**

Case 1: A 60-year-old male was hospitalized after a traffic accident with multiple injuries, including a pseudoaneurysm of the aortic arch. Over an 8-day interval, its size expanded from 3.7 × 2.5 cm to 6.6 × 6.0 × 4.8 cm. He underwent TEVAR but developed postoperative dysphagia and died suddenly at home 22 days after discharge due to massive hematemesis. Case 2: A 53-year-old male presented with acute chest and back pain accompanied by lethargy. Imaging revealed a giant thoracic aortic aneurysm (10.8 × 9.2 × 16.1 cm) compressing the esophagus and trachea. Emergency TEVAR was performed, but he returned 50 days later with cough, fever, dyspnea, and severe anemia.

**Diagnoses:**

Both patients were confirmed to have AEF. In Case 1, autopsy revealed a 3.5 × 2.0 cm mid-esophageal rupture. Case 2 was diagnosed via computed tomography angiography, which showed a 5.0 cm fistulous tract between the aorta and esophagus.

**Interventions:**

Both patients underwent emergency TEVAR using fenestrated stent grafts. After AEF development, Case 2 received transfusions (8 units of packed red blood cells) and broad-spectrum antibiotics but declined further surgical intervention. Case 1 did not receive any intervention prior to his death.

**Outcomes:**

Both patients died from exsanguination due to AEF: Case 1 at 22 days and Case 2 at 50 days after initial surgery. Neither patient underwent definitive surgical repair of the fistula.

**Lessons:**

AEF should be suspected in TEVAR patients with risk factors like large aneurysms or mediastinal hematoma, especially if new symptoms such as dysphagia or fever appear weeks later. Prompt CT angiography is critical. Non-operative management is fatal, and only aggressive surgery offers a potential cure. Prevention requires meticulous stent sizing and measures to reduce infective and mechanical risks.

## 1. Introduction

An aortoesophageal fistula (AEF) is an abnormal communication between the aorta and esophagus, characterized by sudden life-threatening gastrointestinal hemorrhage and severe mediastinal infection. The condition is insidious in onset, rapidly progressive, and carries a high mortality rate of 70% to 90%.^[1]^ With the increasing application of thoracic endovascular aortic repair (TEVAR),^[2]^ post-TEVAR AEF has emerged as a rare but fatal complication requiring heightened clinical vigilance.^[3]^ This study retrospectively analyzed 2 cases of AEF following thoracic aortic stent-graft implantation at our institution over the past 2 years. We systematically investigated the etiology, pathophysiological mechanisms, clinical manifestations, and management strategies of AEF, while summarizing critical lessons learned from these cases to provide clinical warnings and references.

## 2. Cases presentation

### 2.1. Case 1

**Figure 1.**
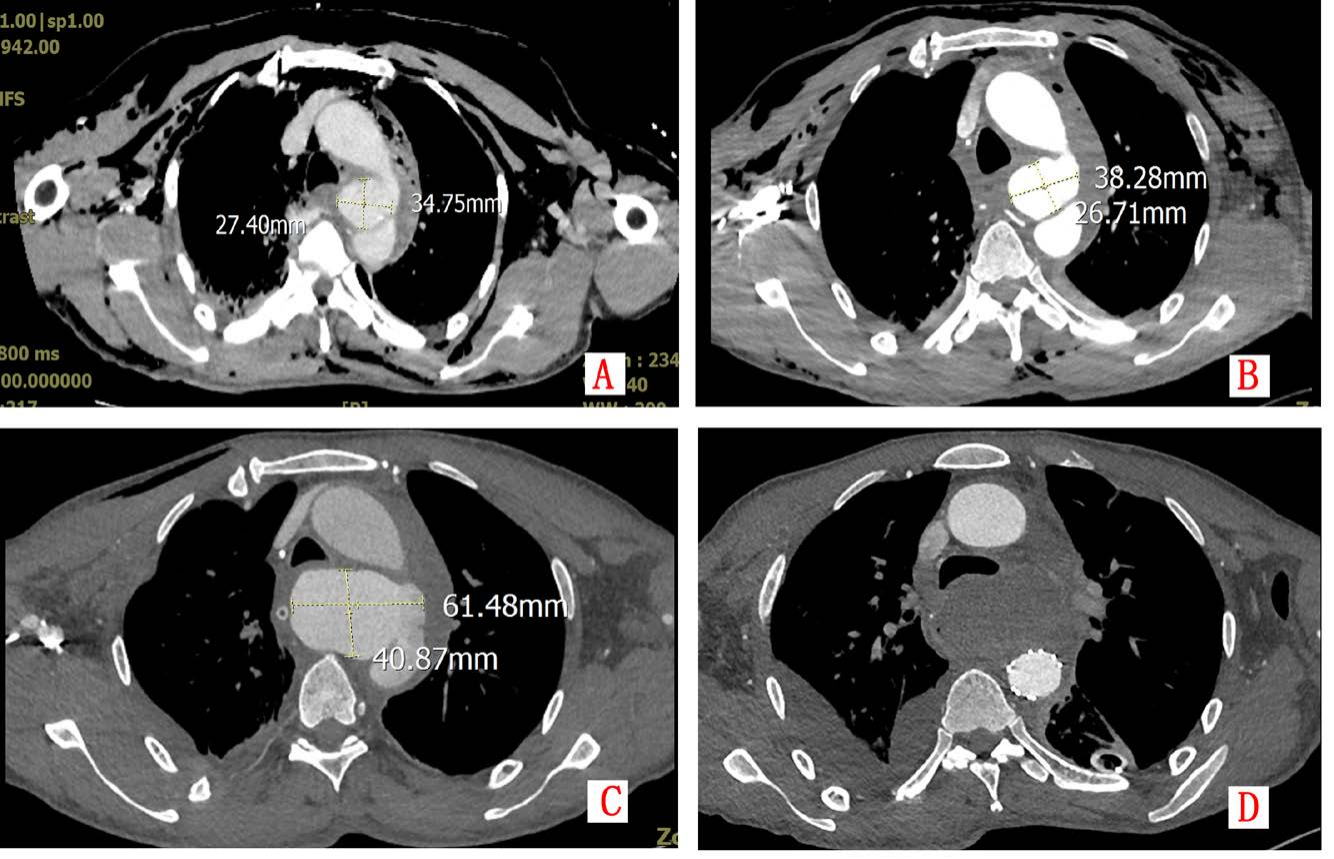
(A–C) Contrast-enhanced CT demonstrates progressive enlargement of a pseudoaneurysm; (D) post-stent implantation imaging reveals stable positioning without contrast leakage.

**Figure 2.**
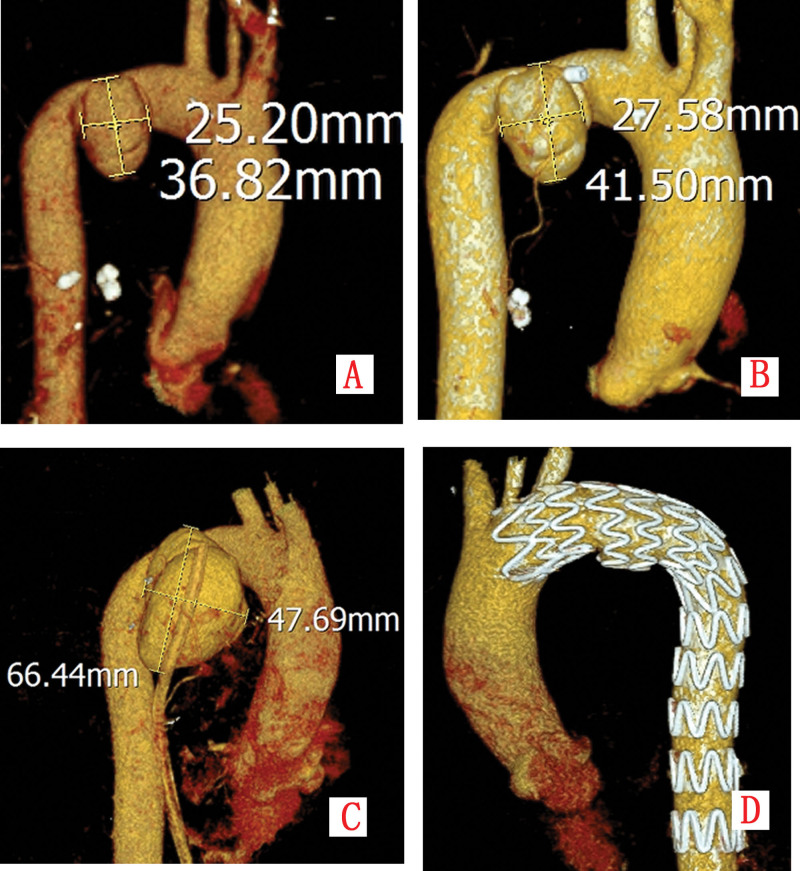
(A–C) Three-dimensional CTA reconstruction illustrates sequential expansion of the pseudoaneurysm; (D) post-TEVAR imaging confirms intact stent morphology and aneurysm exclusion.

A previously healthy 60-year-old male was transferred to our institution in October 2023, approximately 50 hours after a high-energy traffic accident. He presented with generalized pain and respiratory distress. Initial physical examination revealed tachycardia (heart rate 118 bpm), hypotension (90/60 mm Hg), tachypnea (28 breaths/min), and oxygen saturation of 88% on room air. Subcutaneous emphysema was palpated throughout the chest wall and neck, and auscultation demonstrated diminished breath sounds bilaterally. Emergency computed tomography (CT) with angiography revealed severe polytrauma: bilateral nasal bone and nasal septum fractures, left C7 transverse process fracture, multiple bilateral rib fractures (3rd–9th), bilateral hemopneumothorax, pulmonary contusions, mediastinal hematoma with effusion, extensive subcutaneous emphysema, and a 3.7 × 2.5 cm saccular pseudoaneurysm arising from the aortic arch (Fig. 1A and Fig. 2A).

Due to progressive respiratory failure, the patient was intubated and transferred to the ICU. Bilateral thoracic drains were inserted (initial output 600 mL bloody fluid), and intravenous antibiotic therapy with cefazolin (1 g q8 h) was initiated. On hospital day 3, respiratory function improved permitting extubation. Repeat thoracic computed tomography angiography (CTA) showed pseudoaneurysm enlargement to 4.1 × 2.8 cm with increased mediastinal hematoma and new pericardial effusion (Fig. 1B and Fig. 2B). Endovascular repair was recommended but declined by the family. On hospital day 7, the patient developed severe chest pain. Emergency CTA revealed rapid expansion of the pseudoaneurysm to 6.6 × 6.0 × 4.8 cm with irregular contours, indicating impending rupture (Fig. 1C and Fig. 2C). After renewed consultation, the family consented to intervention.

**Figure 3.**
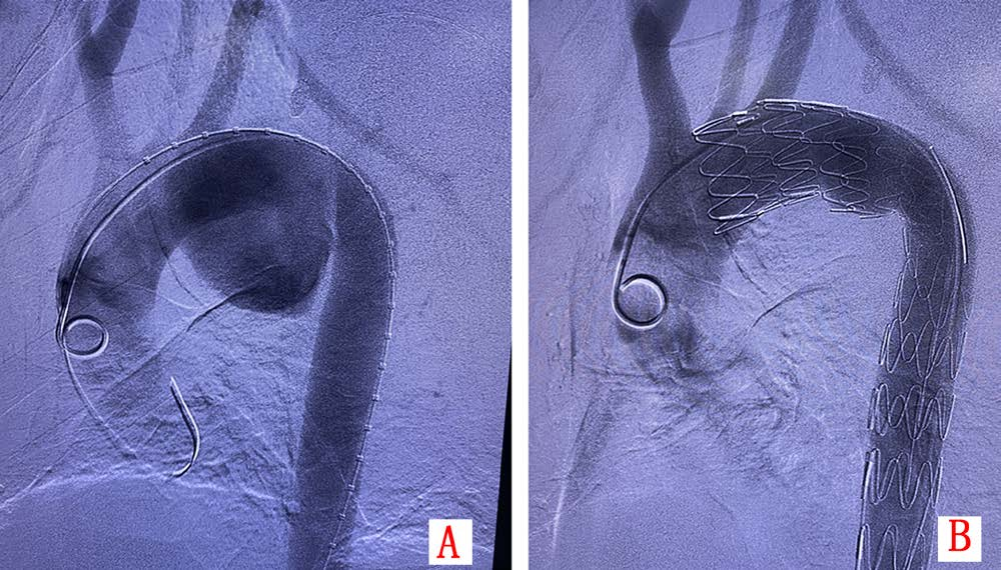
(A) Preoperative aortic angiography visualizes a large pseudoaneurysm; (B) post-stent deployment angiography shows complete exclusion of the pseudoaneurysm and patent left subclavian artery flow.

Emergency endovascular repair was performed. Aortic angiography defined a pseudoaneurysm with a 2-cm neck and 6-cm depth (Fig. 3A). A fenestrated aortic stent graft (TAA3024B200, LifeTech Scientific Co., Ltd., Shenzhen) was deployed, excluding the pseudoaneurysm while preserving left subclavian artery patency (Fig. 3B). Postoperative day 5 CTA confirmed stable stent position, complete exclusion without endoleak, and unchanged mediastinal hematoma (Fig. 1D and Fig. 2D). The postoperative course was complicated by dysphagia managed with enteral nutrition via nasogastric tube and parenteral support. Symptoms gradually resolved, and the patient was discharged on postoperative day 20 tolerating oral diet.

**Figure 4.**
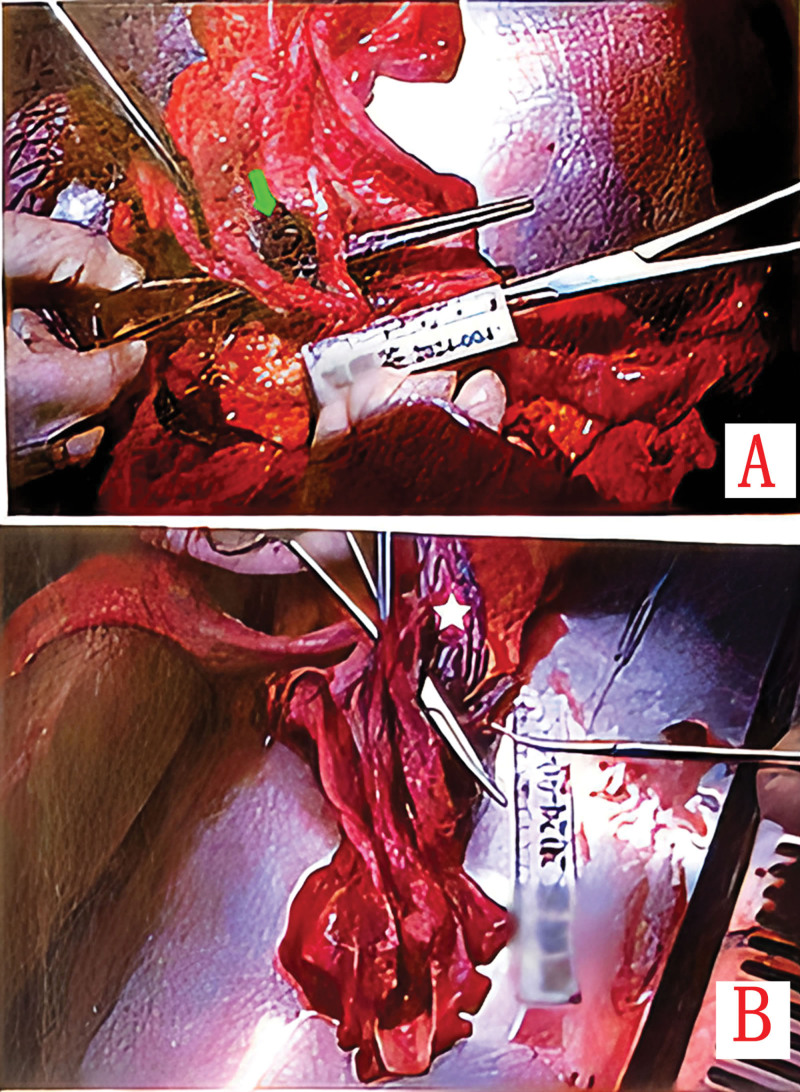
(A) Autopsy identifies a full-thickness esophageal fistula (green arrow) communicating with the aortoesophageal space, demonstrated by forceps traversing the defect; (B) forceps inserted through the fistula confirm continuity with the aortic lumen, highlighting the stent graft (white pentagram).

Twenty-two days after discharge, the patient died suddenly at home following massive hematemesis. Autopsy revealed a 3.5 × 2.0 cm full-thickness esophageal rupture in the mid-esophagus with transmural necrosis. The aortoesophageal space contained necrotic tissue and hematoma, with histology showing esophageal ulceration extending to the aortic media and focal intimal necrosis (Fig. 4A and B).

### 2.2. Case 2

**Figure 5.**
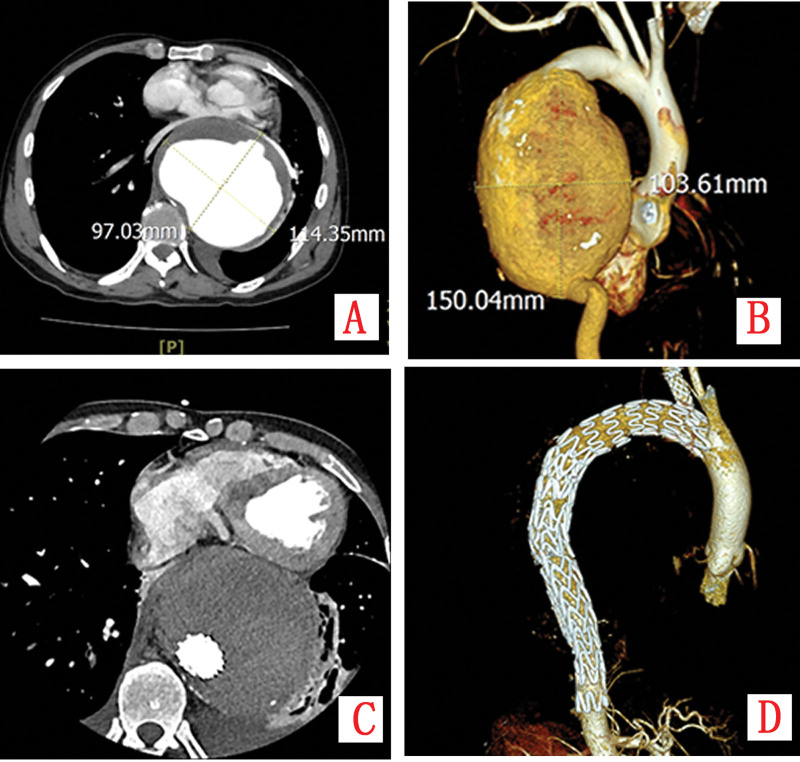
(A, B) CTA reveals a giant fusiform thoracic aortic aneurysm measuring 10.8 × 9.2 × 16.1 cm with peripheral calcification, compressing the esophagus, trachea, and left hilum, accompanied by minimal left pleural effusion without contrast extravasation; (C, D) post-TEVAR CTA demonstrates perigraft thrombosis and absence of endoleak.

A 53-year-old male with hypertension presented to the emergency department in January 2025 with acute-onset, severe tearing pain in his chest and upper back, accompanied by palpitations, dizziness, and lethargy. Initial vitals showed tachycardia (110 bpm) and hypotension (85/50 mm Hg). Emergency CTA revealed a giant fusiform thoracic aortic aneurysm (10.8 × 9.2 × 16.1 cm) with peripheral calcification, causing significant compression of the esophagus, trachea, and left pulmonary hilum. A small left pleural collection (22 HU) was noted without contrast extravasation, suggestive of contained rupture (Fig. 5A and B).

**Figure 6.**
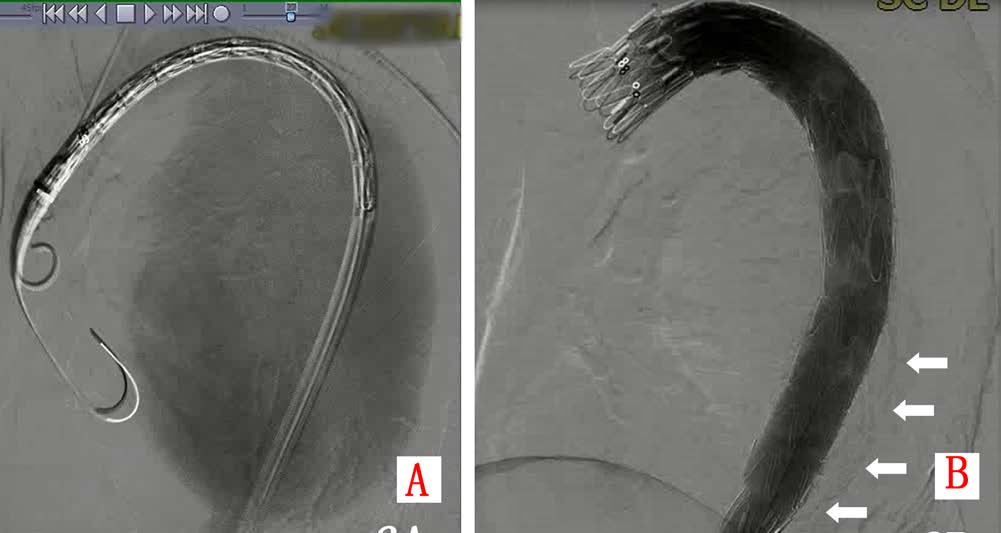
(A) Aortic angiography delineates a massive thoracic aortic aneurysm; (B) a cluster of white arrows indicates a small amount of contrast medium extravasation at the distal end of the stent, suggesting a Type Ib endoleak.

The patient underwent emergency TEVAR. Aortic angiography confirmed the aneurysm morphology (Fig. 6A). Procedure involved: deployment of a fenestrated stent graft (TAA2622B160, LifeTech Scientific Co., Ltd., Shenzhen) with fenestration aligned to the left subclavian artery; placement of a 2nd stent graft (TAA2420B160, LifeTech Scientific Co., Ltd., Shenzhen) 5 cm distally; management of type Ib endoleak (Fig. 6B) with a 3rd stent graft (TAA2420B120, LifeTech Scientific Co., Ltd., Shenzhen); revascularization of the left subclavian artery with balloon angioplasty and additional stent graft (10 × 40 mm). Final angiography showed successful exclusion without endoleak. Postoperative day 2 CTA demonstrated no endoleak but extensive perigraft thrombosis (Fig. 5C and D). The patient was discharged on postoperative day 10.

**Figure 7.**
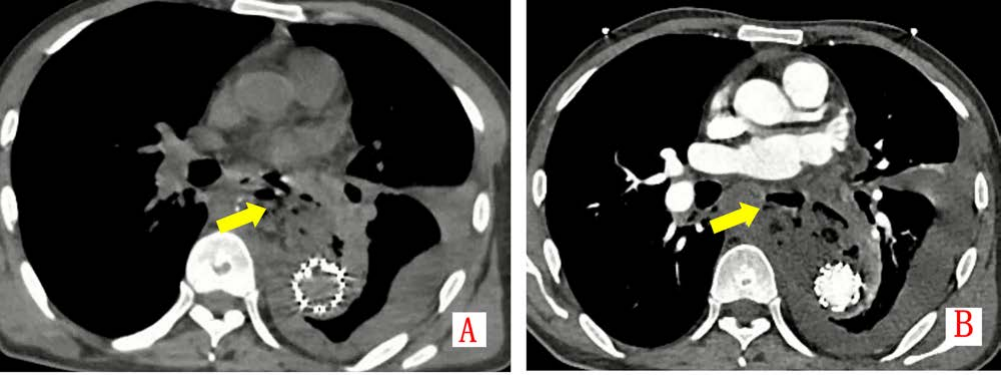
(A, B) Non-contrast CT and CTA exhibit flocculent hyperdensities, gas accumulation around the stent, and a 5.0 cm fistulous tract connecting to the esophagus, consistent with infected aorto-esophageal fistula, alongside moderate left encapsulated hemothorax.

On postoperative day 50, he represented with fever (38.8°C), cough, and dyspnea. Thoracic CT/CTA revealed gas and fluid collections around the stent graft with a 5.0 cm fistulous tract to the esophagus, confirming aortoesophageal fistula (Fig. 7A and B). Laboratory studies showed severe anemia (hemoglobin 38 g/L, reference 130–175 g/L). Management included transfusion (8 units PRBC), intravenous antibiotics (cefoperazone-sulbactam 3 g q12 h), and parenteral nutrition. The patient declined surgical intervention and was discharged against medical advice. He died 5 days later from massive hematemesis.

## 3. Discussion

AEF after TEVAR is an uncommon but devastating complication. As illustrated in the 2 present cases, the outcome is almost invariably fatal. The pathophysiology of secondary AEF is multifactorial, often involving mechanical compression, ischemic injury, and inflammatory processes.^[4]^ Stent grafts can exert persistent stress on the esophageal wall, while perigraft hematoma or residual aneurysm may cause sustained mediastinal compression, compromising the vascular supply to the esophagus. Infectious mechanisms and underlying aortic pathology may also contribute.^[4]^

In both cases, AEF developed within 2 months after TEVAR. Each patient had a large aortic lesion (either pseudoaneurysm or true aneurysm) causing significant esophageal compression. Although emergency TEVAR was performed, unresolved mediastinal hematoma and stent-induced pressure likely resulted in persistent esophageal ischemia, leading to necrosis and eventual fistula formation. The delayed onset of AEF (22 and 50 days after discharge, respectively) suggests a subacute process dominated by chronic compression and microvascular compromise rather than acute intraoperative injury. This aligns with previous reports indicating that even technically successful TEVAR does not eliminate the risk of AEF in patients with large aneurysms or significant mediastinal hematoma.

AEF classically presents with Chiari triad,^[5]^ yet diagnosis is often delayed due to nonspecific symptoms and infrequent follow-up in resource-limited settings. Contrast-enhanced CT has high sensitivity for AEF,^[3]^ typically showing perigraft gas, mediastinal fluid, or contrast extravasation: though thrombosed fistulas may not exhibit extravasation. Esophagogastroduodenoscopy is diagnostic but carries a high risk of iatrogenic hemorrhage,^[6]^ and was not performed in our patients due to their critical condition or postmortem diagnosis.

It is noteworthy that neither patient presented with prominent pain upon readmission, which contributed to the diagnostic delay. Acute aortic syndromes, including AEF and impending rupture, can occasionally present with minimal or no pain, a phenomenon that is underrecognized yet not uncommon in clinical practice.^[7,8]^ This underscores the need for heightened clinical suspicion even in the absence of classic symptoms.

Management of AEF remains extremely challenging. Conservative treatment is uniformly fatal,^[9,10]^ and even with operative intervention (including open repair with graft explantation and esophageal reconstruction) reported mortality exceeds 50%.^[11–17]^ Both patients in this report died despite maximal medical support, reflecting the grave prognosis once AEF develops. These cases emphasize that patients with large aortic aneurysms, perioperative hematoma, or persistent symptoms such as dysphagia after TEVAR should be monitored closely. Early imaging may be considered in high-risk cases, though there is no consensus on optimal surveillance strategies.

This study has several notable limitations. First, as a retrospective case series of only 2 patients, our report lacks statistical power and is subject to the inherent biases of such a design. The findings and conclusions are descriptive and hypothesis-generating rather than definitive. Second, the generalizability of our observations may be limited, as both cases presented with exceptionally large aortic lesions and massive mediastinal hematomas, which represent a high-risk subset of TEVAR patients. Third, as highlighted in the discussion, the diagnosis of AEF was postmortem in 1 case and delayed in the other due to the absence of classic symptoms like pain upon readmission, underscoring the challenge of timely diagnosis in real-world practice. Finally, and most significantly, the fatal outcome in both cases precludes any evaluation of the efficacy of potential therapeutic interventions, such as aggressive surgical repair, for managing this devastating complication at our institution.

## 4. Conclusion

AEF following TEVAR is a devastating complication with a multifactorial pathophysiology involving mechanical stress, ischemia, and infection. Our report, limited to 2 fatal cases, does not aim to redefine management paradigms but rather to highlight that this condition can develop insidiously and prove fatal despite initial technical success. Importantly, clinicians should be aware that acute aortic pathologies can sometimes evolve without typical pain, delaying diagnosis. A high index of suspicion, structured follow-up, and prompt cross-sectional imaging are essential in high-risk patients, though currently, outcomes remain poor. Future efforts should focus on earlier detection and multidisciplinary strategies to mitigate this rare but lethal complication.

## Author contributions

**Conceptualization:** Zhi Wen, Changxue Wu.

**Data curation:** Zhi Wen, Zheng Wang, Jianqiang Wu, Mingwu Tian.

**Formal analysis:** Jianqiang Wu, Changxue Wu.

**Investigation:** Zheng Wang.

**Supervision:** Changxue Wu.

**Writing – original draft:** Zhi Wen, Mingwu Tian

**Writing – review & editing:** Zhi Wen, Changxue Wu
